# Test–Retest Reliability of Postural Sway Measures Using a Portable Low‐Cost Force Plate in Healthy Adults

**DOI:** 10.1002/pri.70270

**Published:** 2026-07-02

**Authors:** Miha Vindiš, Žiga Kozinc, Nenad Nedović

**Affiliations:** ^1^ Faculty of Health Sciences University of Primorska Izola Slovenia; ^2^ Ludwig Boltzmann Institute for Rehabilitation Research Vienna Austria; ^3^ Academy of Applied Studies Belgrade College of Health Sciences Belgrade Serbia; ^4^ Institute for Medical Research University of Belgrade Belgrade Serbia

**Keywords:** balance, center of pressure, postural control, reliability

## Abstract

**Background and Purpose:**

Center of pressure (CoP) metrics derived from force plates are widely used to quantify postural control, but laboratory‐grade systems limit routine clinical and field implementation. Portable low‐cost force plates could enable physiotherapists to monitor balance longitudinally, provided that their measurements are sufficiently reliable and clinically interpretable. This study addresses a gap in the literature by systematically comparing the test–retest reliability of CoP outcomes across bipedal and single‐leg stance conditions using a portable low‐cost force plate, providing device‐specific and task‐specific validation data relevant to clinical and field‐based implementation.

**Methods:**

This test–retest reliability study included 27 healthy young adults who completed two laboratory sessions 7–10 days apart. In each session, participants performed three 30‐s trials of bipedal quiet stance and three 30‐s trials of single‐leg stance on each leg barefoot with standardized arm position and visual fixation. Mean values across repetitions were analyzed. Relative reliability was assessed using two‐way mixed‐effects ICC for absolute agreement (3,1), with 95% confidence intervals. Absolute reliability was quantified using typical error (TE) and coefficient of variation (CV). Paired *t*‐tests evaluated systematic between‐session differences.

**Results:**

Across all outcomes, single‐leg stance demonstrated substantially higher inter‐session reliability compared to bipedal stance. Measures of total CoP displacement and velocity during single‐leg stance exhibited good to excellent reliability (ICC = 0.85–0.90) and low absolute error (CV ≈ 8%), with direction‐specific displacements showing similarly consistent results (ICC = 0.83–0.90; CV generally < 10%). In contrast, the CoP ellipse area and standard deviation measures were considerably less stable (ICC = 0.57–0.79) and displayed markedly higher variability (CV ≈ 16%–23%). The contrast was even more pronounced in the bipedal stance, where reliability was poor to moderate across all parameters (ICC = 0.39–0.65), with the ellipse area exhibiting excessive variability (CV > 60%). Notably, no systematic between‐session differences were observed for most outcomes, further supporting the consistency of the measurements.

**Discussion:**

In healthy young adults, single‐leg stance provides more reliable CoP measures than bipedal quiet stance when using a portable low‐cost force plate. Displacement‐ and velocity‐based outcomes during single‐leg stance appear most suitable for repeated assessments and monitoring. MDC_95_ values should be interpreted strictly as measurement‐error thresholds and not as indicators of clinical responsiveness or meaningful clinical change because responsiveness was not evaluated.

## 1 Introduction

The quantification of postural sway through center of pressure (CoP) trajectory analysis has become a fundamental methodology in postural control assessment (Palmieri et al. 2002). Postural sway reflects the continuous, subconscious adjustments made by the neuromuscular system to maintain upright balance, offering insight into the integrity of sensorimotor integration and motor output (Rogers and Mille 2018). Quantifying these subtle oscillations through CoP metrics provides a sensitive and non‐invasive means of assessing balance function across diverse populations, from healthy children and athletes to individuals with neurological or musculoskeletal conditions (Richmond et al. 2021; Makaracı et al. 2022; Melton et al. 2024; Makaraci, Nas, Gunduz, and Ileri 2024). A substantial body of research demonstrates that CoP parameters such as path length or mean velocity generally exhibit robust test‐retest reliability, although results can vary across stance conditions, sensory manipulations (e.g., eyes‐open vs. eyes‐closed), and studied populations (Dallinga et al. 2012; Johansson et al. 2017; Dobberke et al. 2022; Campolettano et al. 2020; Karimi Ghasem Abad et al. 2021). Challenging conditions sometimes yield higher reliability, likely because increased task demands expand between‐subject variability relative to measurement error, thereby improving the signal‐to‐noise ratio of postural sway measures (Dobberke et al. 2022; Campolettano et al. 2020). This is a principle rooted in classical measurement theory, where ICC reflects the proportion of total variance attributable to true between‐subject differences (Shrout and Fleiss 1979). A higher signal‐to‐noise ratio, achieved through greater postural demand, thus favors more stable and discriminative relative reliability estimates.

However, translating these reliable laboratory methods into everyday clinical or field environments remains challenging due to the dependence on expensive, laboratory‐grade equipment. This limitation has facilitated the development and validation of more affordable and portable alternatives, with studies like Lo et al. (2022) demonstrating that low‐cost, custom‐built plates can achieve reliability comparable to commercial systems even under standardized testing conditions (e.g., fixed gaze on a target, controlled stance width) but also revealed an age‐dependent reliability of sway metrics, with older adults showing more reliable anteroposterior measures and younger individuals superior mediolateral reliability, underscoring the need for population‐specific assessment protocols. This finding is supported by other research on consumer‐grade technology, such as an HTC Vive VR tracker, which showed acceptable reliability and good convergent validity in distinguishing between different stance and visual conditions (Liang et al. 2022), and dedicated portable devices such as the Tymo balance plate, which demonstrated a very strong correlation with laboratory standards (Meier et al. 2025). Even the low‐cost Nintendo Wii Balance Board (WBB) shows excellent concurrent validity for measuring CoP, though its reliability can be variable (Vredeveld et al. 2025). These findings collectively underscore a pivotal shift towards accessible and practical technologies, enabling the reliable assessment of postural sway under more challenging conditions beyond the limits of well‐funded laboratories and into broader clinical settings.

From a measurement perspective, understanding how task constraints influence the reliability of postural sway outcomes is essential for selecting appropriate balance assessments in sport and exercise science. This need is particularly relevant given the growing use of portable, low‐cost force plates, which have been increasingly adopted in clinical and field‐based settings due to their accessibility. However, despite their widespread application, comprehensive reliability data for such devices remain limited, especially across different stance conditions. To address this gap, the aim of this study is to examine the test–retest reliability of various CoP parameters during bipedal and single‐leg stance using a portable low‐cost force plate. The novelty of this study lies in the systematic evaluation of test–retest reliability of postural sway measurements obtained with a specific portable low‐cost force plate (EasyBase, Meloq AB), directly comparing bipedal and single‐leg stance conditions while reporting both relative and absolute reliability indices (a combination not previously addressed for this device). Based on previous literature examining stance conditions and outcome measures, we hypothesize that: (1) single‐leg stance will demonstrate greater relative and absolute reliability compared to bipedal stance due to the higher neuromuscular demand and reduced base of support; and (2) velocity‐based parameters will exhibit superior reliability compared to area‐based metrics, which are more susceptible to trial‐to‐trial variability.

## 2 Methods

### 2.1 Participants

A total of 27 healthy adults took part in the study (21 men and 6 women; age 22.25 ± 2.7 years; height 1.79 ± 0.17 m; body mass 76.80 ± 9.11 kg). Recruitment was conducted via convenience sampling among students of the University of Primorska and individuals from the surrounding community using online announcements and personal referrals. All participants were free of acute or chronic musculoskeletal problems and reported no previous balance‐related conditions. Before enrollment, they were informed in detail about the study aims and procedures and provided written informed consent. To control for the potential influence of fatigue, they were asked to avoid vigorous resistance training for 72 h and any structured exercise for 24 h preceding each testing visit. The study protocol, which involved only non‐invasive assessments, was approved by the University of Primorska’s Commission for Ethics in Human Subjects Research (approval No. 4264‐19‐6/23).

### 2.2 Study Design and Procedures

**FIGURE 1:**
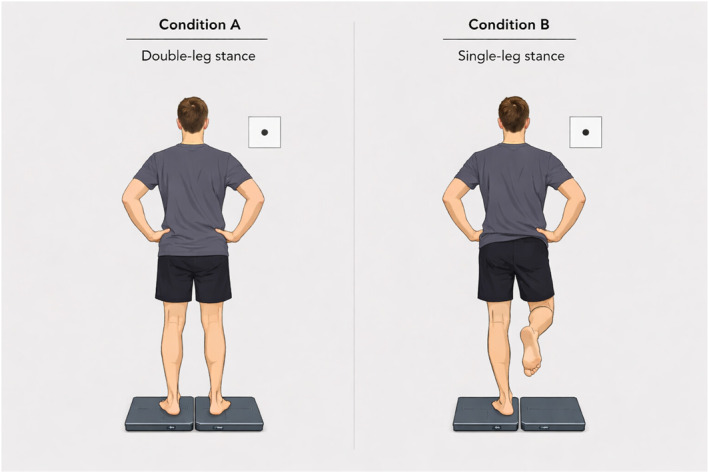
The study conditions involved bipedal quiet stance (condition A) and single‐leg quiet stance (condition B).

This study used a repeated‐measures design to evaluate inter‐session reliability of postural sway parameters during bipedal and single‐leg stance tasks. Participants completed two laboratory sessions scheduled 7–10 days apart, with all assessments performed under comparable environmental conditions and at the same time of day to minimize diurnal fluctuations. The same examiner conducted all measurements using identical equipment, setup, and instructions across both sessions, and no performance‐related feedback was provided at any point. During each visit, participants performed a series of quiet‐standing balance tasks on a portable force plate. The testing protocol was randomized and included three 30‐s bipedal stance trials and three 30‐s single‐leg trials on each leg (Figure 1). All trials were performed barefoot. For single‐leg stance, the non‐stance limb was flexed at the knee and held without touching the stance leg, while the stance leg remained extended but not hyperextended. In all conditions, participants were instructed to stand as still as possible with their hands placed on their hips. To standardize visual input, they fixed a stationary target placed at eye level approximately 3 m away.

Before the first recorded trial of each stance condition, a single familiarization attempt was made to ensure proper execution. Rest intervals of 60 s were given between trials and avoid unnecessary fatigue. The randomized sequence of tasks was repeated during the second session. A 30‐s trial duration was selected to balance the need for sufficient signal stability with the practical considerations of participant burden, especially during single‐leg stance. Although longer trials may improve reliability for some CoP outcomes, prior work suggests that 30‐s recordings remain adequate for most commonly used sway parameters in both research and applied settings (Pinsault and Vuillerme 2009).

### 2.3 Data Processing and Outcome Variables

Ground reaction force data were collected using a portable low‐cost force plate system (EasyBase, Meloq AB, Sweden). The system consists of two independent wireless force plates with each one incorporating four strain‐gauge load cells measuring vertical ground reaction forces, from which CoP coordinates in the anteroposterior and mediolateral directions are derived using standard moment equations. The plates feature an aluminum top surface with foot positioning markers to enhance standardization across trials. The plate dimensions are approximately 40 × 40 cm, with a reported measurement accuracy within 1% by the manufacturer. The device weighs approximately 2 kg, enabling easy transport and field deployment without requiring fixed installation or external amplification hardware. At an approximate retail price of 2000–2500 EUR, it represents a substantially more accessible alternative to laboratory‐grade force plates, which typically range from 10,000 to 30,000 EUR or more (Miller et al. 2023). Its wireless design and compact form factor further support its classification as portable, requiring no fixed installation or external amplification hardware.

Signals were sampled at 400 Hz and processed using the manufacturer's proprietary software. The investigators did not independently process raw force/load‐cell data, reconstruct CoP coordinates, or apply custom filtering procedures. Instead, the CoP‐derived outcome variables reported in this study were exported directly from the manufacturer's system. Therefore, filtering, CoP coordinate calculation, and velocity estimation were embedded within the proprietary device–software pipeline and were not directly controlled or modified by the investigators. According to the manufacturer's documentation, the software follows the computational conventions described in Quijoux et al. (2021), including low‐pass filtering of CoP signals and velocity estimation procedures. However, because these procedures are implemented within proprietary software, the present study evaluates the reliability of the EasyBase device–software system as used in practice, rather than an independently implemented raw‐signal processing workflow.

For each 30‐s trial, the software generated a set of CoP‐derived parameters. The following variables were retained for analysis:

- Total CoP Displacement (mm): the cumulative length of the two‐dimensional CoP path over the trial.
- Displacement Velocity (mm/s): mean velocity of the CoP, computed as total displacement divided by trial duration.
- CoP Ellipse Area (mm^2^): the 95% confidence ellipse area encompassing the two‐dimensional CoP trajectory, reflecting the overall sway magnitude.
- Standard Deviation *X*/*Y* (mm): variability of the CoP position in the mediolateral (*X*) and anteroposterior (*Y*) directions.
- AP CoP Displacement (mm): cumulative distance traveled along the anteroposterior axis.
- ML CoP Displacement (mm): cumulative distance traveled along the mediolateral axis.

Full mathematical definitions and computational procedures for all CoP variables reported in this study follow the conventions described in Quijoux et al. (2021), which provides an open‐access compendium of standardized algorithms for stabilogram‐derived variables, including explicit formulas and reproducible code (available at https://github.com/Jythen/code_descriptors_postural_control). However, these algorithms were not independently implemented by the investigators. The reported parameters reflect the output of the manufacturer's software, and reproducibility of the present protocol therefore depends on using the same device–software system and export procedures.

Specifically, the 95% confidence ellipse area was derived from the covariance matrix of the two‐dimensional centered CoP trajectory, with *χ* ^2^ scaling applied via the 0.95‐quantile of the Fisher distribution with 2 and *n* − 2 degrees of freedom, yielding:

$$\text{Area}=\pi \times F_{0.95,2,n-2}\times \surd \left(\lambda_{1}\times \lambda_{2}\right),$$

where *λ*_1_ and *λ*_2_ are the eigenvalues of the covariance matrix. Total CoP displacement was computed as the cumulative Euclidean distance between consecutive CoP sample points, and displacement velocity as total displacement divided by trial duration. Direction‐specific displacements (AP and ML) were computed as the cumulative distances traveled along each axis independently. Standard deviations in the *X* and *Y* directions represent the square root of the variance of the centered CoP position along each respective axis.

For each participant and stance condition, the mean value of three repetitions was used for all intra‐session and inter‐session reliability analyses, with trial averaging applied to reduce the influence of within‐session variability on reliability estimates. Because no simultaneous comparison with a laboratory‐grade force plate was performed, the present study should be interpreted as an evaluation of test–retest reliability of the EasyBase device–software system and not as evidence of criterion validity or cross‐device agreement.

### 2.4 Statistical Analysis

Statistical analyses were performed using SPSS Statistics for Windows, version 26.0 (IBM Corp., Armonk, NY, USA) and Microsoft Excel (Microsoft Corp., Redmond, WA, USA). Descriptive statistics are expressed as means ± standard deviation. Paired sample *t*‐tests were conducted to examine systematic differences between sessions. For each variable, the mean difference with its 95% CI and the corresponding *p*‐value is reported. Relative reliability was assessed using an intra‐class correlation coefficient (ICC) with two‐way mixed‐effects model for absolute agreement (model 3,1). A two‐way mixed‐effects model for absolute agreement was selected, as the same raters and measurement system were used across sessions and inference was limited to these conditions. ICC was interpreted in line with commonly accepted thresholds—values below 0.50 were considered indicative of poor reliability, 0.50–0.75 as moderate, 0.75–0.90 as good, and values above 0.90 as excellent (Koo and Li 2016). Ninety‐five percent confidence intervals (95% CI) were calculated to assess the precision of each ICC estimate. To assess absolute reliability, we calculated TE with 95% CI, which represents the standard deviation of the test–retest differences divided by √2. The CV based on the TE was also computed by expressing TE as a percentage of the grand mean across both sessions (Hopkins 2000), with CV < 10% considered to be acceptable. The CV < 10% threshold proposed by Hopkins (2000) was adopted as a practical benchmark for acceptable absolute reliability. Although this criterion originates from sport science rather than posturography specifically, it has been applied in comparable balance reliability studies (Meshkati et al. 2011; Kozinc et al. 2025) and is used here descriptively alongside ICC and MDC_95_ rather than as a definitive decision criterion. Minimal Detectable Change at the 95% confidence level (MDC_95_) was subsequently derived as SEM × 1.96 × √2, representing the smallest change exceeding measurement error with 95% confidence (Makaraci et al. 2024). Statistical significance was set at *p* < 0.05.

## 3 Results

Table 1 shows descriptive statistics and mean differences between visits. The corresponding reliability indices (ICC, TE, and CV) for all center‐of‐pressure parameters across stance conditions are shown in Table 2. Paired‐sample *t*‐tests showed no systematic between‐session differences for the majority of variables, with all *p*‐values ≥ 0.057. The only exception was bipedal standard deviation in the mediolateral direction, where Visit 1 values were higher than Visit 2 (mean difference = 0.14 mm, 95% CI 0.03–0.25 mm, *p* = 0.012).

**TABLE 1:** Means ± SD for Visit 1 and Visit 2 across stance conditions, with between‐session mean differences (MD) and 95% CI.

Variable	Condition	Visit 1 mean ± SD	Visit 2 mean ± SD	MD (95% CI)	*p*‐value
Total CoP displacement (mm)	Bipedal	198.85 ± 55.95	190.83 ± 51.54	8.02 (−14.67 to 30.72)	0.473
	Single‐leg left	1210.81 ± 249.90	1163.75 ± 253.49	47.06 (−10.43 to 104.55)	0.104
	Single‐leg right	1312.27 ± 313.41	1265.60 ± 316.37	46.67 (−11.22 to 104.56)	0.109
Displacement velocity (mm/s)	Bipedal	6.41 ± 1.80	6.16 ± 1.66	0.26 (−0.47 to 0.99)	0.473
	Single‐leg left	39.06 ± 8.06	37.54 ± 8.18	1.52 (−0.34 to 3.37)	0.104
	Single‐leg right	42.33 ± 10.11	40.83 ± 10.21	1.51 (−0.36 to 3.37)	0.109
CoP ellipse area (mm^2^)	Bipedal	37.78 ± 32.87	25.81 ± 22.27	11.97 (−0.41 to 24.35)	0.057
	Single‐leg left	1008.07 ± 267.40	935.92 ± 292.84	72.16 (−19.29 to 163.61)	0.117
	Single‐leg right	1058.33 ± 574.33	1042.73 ± 434.35	15.60 (−120.82 to 152.02)	0.816
Standard deviation *X* (mm)	Bipedal	0.50 ± 0.31	0.36 ± 0.13	**0.14 (0.03 to 0.25)**	**0.012**
	Single‐leg left	6.68 ± 0.90	6.50 ± 1.04	0.18 (−0.18 to 0.54)	0.316
	Single‐leg right	6.72 ± 1.36	6.69 ± 1.27	0.04 (−0.39 to 0.47)	0.859
Standard deviation *Y* (mm)	Bipedal	4.41 ± 1.41	4.15 ± 2.00	0.25 (−0.40 to 0.91)	0.431
	Single‐leg left	8.13 ± 1.45	7.67 ± 1.44	0.46 (−0.09 to 1.02)	0.098
	Single‐leg right	8.24 ± 2.52	8.18 ± 2.23	0.06 (−0.58 to 0.70)	0.854
AP CoP displacement (mm)	Bipedal	194.01 ± 53.22	186.77 ± 50.73	7.24 (−14.82 to 29.30)	0.505
	Single‐leg left	717.30 ± 170.41	689.04 ± 166.53	28.26 (−12.95 to 69.48)	0.170
	Single‐leg right	795.20 ± 237.83	768.78 ± 219.45	26.41 (−17.59 to 70.42)	0.228
ML CoP displacement (mm)	Bipedal	25.63 ± 14.30	23.00 ± 8.17	2.63 (−1.40 to 6.67)	0.191
	Single‐leg left	829.58 ± 169.11	797.87 ± 180.63	31.71 (−9.64 to 73.06)	0.127
	Single‐leg right	882.04 ± 176.08	851.17 ± 197.45	30.86 (−4.64 to 66.36)	0.086

*Note:* Bold indicates statistically significant systematic difference between visits.

**TABLE 2:** Reliability indices (ICC with 95% CI, typical error, and coefficient of variation) across stance conditions.

Variable	Condition	ICC (95% CI)	TE (95% CI)	CV % (95% CI)	MDC
Total CoP displacement (mm)	Bipedal	0.47 (0.11–0.72)	39.74 (31.16–54.85)	20.39 (15.99–28.15)	110.1
	Single‐leg left	0.85 (0.70–0.93)	100.65 (78.93–138.93)	8.48 (6.65–11.70)	278.8
	Single‐leg right	0.90 (0.80–0.96)	101.35 (79.48–139.90)	7.86 (6.17–10.85)	280.8
Displacement velocity (mm/s)	Bipedal	0.47 (0.11–0.72)	1.28 (1.01–1.77)	20.39 (15.99–28.15)	3.55
	Single‐leg left	0.85 (0.70–0.93)	3.25 (2.55–4.48)	8.48 (6.65–11.70)	9.00
	Single‐leg right	0.90 (0.80–0.96)	3.27 (2.56–4.51)	7.86 (6.17–10.85)	9.06
CoP ellipse area (mm^2^)	Bipedal	0.42 (0.05–0.69)	21.67 (17.00–29.91)	68.16 (53.46–94.09)	60.1
	Single‐leg left	0.69 (0.42–0.85)	160.10 (125.56–221.00)	16.47 (12.92–22.74)	443.5
	Single‐leg right	0.79 (0.59–0.90)	238.83 (187.30–329.68)	22.73 (17.83–31.38)	661.6
Standard deviation *X* (mm)	Bipedal	0.39 (0.01–0.67)	0.19 (0.15–0.26)	43.98 (34.49–60.71)	0.53
	Single‐leg left	0.60 (0.28–0.80)	0.63 (0.49–0.86)	9.50 (7.45–13.11)	1.74
	Single‐leg right	0.69 (0.41–0.85)	0.75 (0.59–1.04)	11.24 (8.82–15.52)	2.08
Standard deviation *Y* (mm)	Bipedal	0.58 (0.25–0.78)	1.15 (0.90–1.59)	26.84 (21.05–37.06)	3.19
	Single‐leg left	0.57 (0.24–0.78)	0.97 (0.76–1.34)	12.28 (9.63–16.95)	2.69
	Single‐leg right	0.79 (0.59–0.90)	1.12 (0.88–1.55)	13.70 (10.74–18.91)	3.10
AP CoP displacement (mm)	Bipedal	0.46 (0.10–0.72)	38.62 (30.29–53.32)	20.29 (15.91–28.00)	106.9
	Single‐leg left	0.83 (0.65–0.92)	72.16 (56.59–99.61)	10.26 (8.05–14.17)	199.8
	Single‐leg right	0.89 (0.78–0.95)	77.04 (60.42–106.35)	9.85 (7.73–13.60)	213.4
ML CoP displacement (mm)	Bipedal	0.65 (0.35–0.83)	7.07 (5.55–9.76)	29.08 (22.81–40.14)	19.58
	Single‐leg left	0.84 (0.68–0.92)	72.39 (56.77–99.93)	8.90 (6.98–12.28)	200.5
	Single‐leg right	0.90 (0.79–0.95)	62.15 (48.74–85.80)	7.17 (5.62–9.90)	172.16

Single‐leg stance demonstrated consistently higher inter‐session reliability compared with bipedal stance. Across the single‐leg conditions, most center‐of‐pressure parameters showed moderate to excellent relative reliability, with ICC values ranging from 0.57 to 0.90. Total CoP displacement and displacement velocity were among the most stable measures, yielding ICC values between 0.85 and 0.90 for both legs, accompanied by comparatively low coefficients of variation (approximately 7%–9%). Direction‐specific displacements in the anteroposterior and mediolateral directions exhibited a similar pattern, with ICC values between 0.83 and 0.90 and CV values generally remaining below 10%. The CoP ellipse area produced more variable results: the left‐leg condition demonstrated only moderate reliability (ICC = 0.69), while the right‐leg condition reached good reliability (ICC = 0.79), although both displayed relatively high absolute variability, as reflected by CV values between 16% and 23%. Standard deviations in both movement directions showed the weakest reliability within single‐leg stance, particularly for the *Y*‐direction on the left leg (ICC = 0.57), whereas right‐leg values were slightly higher, with ICCs rising to 0.79. CV values of 16%–23% for ellipse area and standard deviation measures, and exceeding 60% for bipedal ellipse area indicate that a substantial proportion of between‐session score variation likely reflects measurement noise rather than true change, limiting their utility for longitudinal monitoring.

In contrast, bipedal stance produced markedly lower inter‐session reliability across all parameters. ICC values for bipedal variables ranged from 0.39 to 0.65, indicating poor to moderate consistency. Both total CoP displacement and displacement velocity reached an ICC of only 0.47 and were further characterized by relatively high CV values of around 20%. The CoP ellipse area showed the poorest performance among all bipedal variables, with an ICC of 0.42 and a CV exceeding 60%, indicating substantial between‐session variability. Standard deviations in both the *X* and *Y* directions showed similarly weak reproducibility with ICC values between 0.39 and 0.58. Directional displacements followed the same trend: anteroposterior displacement demonstrated poor to moderate reliability (ICC = 0.46), whereas mediolateral displacement reached only moderate reliability (ICC = 0.65). These findings were mirrored in the absolute reliability indices, where TE and CV values were consistently larger in bipedal conditions compared with single‐leg conditions.

MDC_95_ values further contextualize the absolute reliability of CoP measures by indicating the magnitude of change required to exceed the measurement error with 95% confidence. However, MDC_95_ should not be interpreted as evidence of responsiveness, as the present study did not examine whether these outcomes detect true change following rehabilitation, intervention, or clinical recovery. During single‐leg stance, MDC_95_ for total CoP displacement ranged from 279 to 281 mm, and for displacement velocity from 9.00 to 9.06 mm/s, indicating that changes of this magnitude are necessary to exceed measurement error with 95% confidence. Directional displacements showed MDC_95_ values of approximately 200–213 mm for the anteroposterior and 172–201 mm for the mediolateral direction. In contrast, bipedal stance yielded substantially lower absolute MDC_95_ values for most parameters due to the smaller sway magnitudes inherent to this condition; however, given the poor relative reliability observed, these values should be interpreted cautiously as they do not reflect a sensitive or stable measurement context. The CoP ellipse area showed the largest MDC_95_ values relative to mean scores across both conditions, consistent with its high within‐subject variability. These values should not be interpreted as thresholds for clinically meaningful change, because responsiveness to intervention or clinical recovery was not assessed.

## 4 Discussion

The primary finding of this study is that postural sway measures obtained with a portable low‐cost force plate demonstrate substantially higher inter‐session reliability during single‐leg stance than during bipedal stance. Across most center‐of‐pressure parameters, single‐leg conditions yielded good to excellent relative reliability and acceptable absolute error, whereas bipedal stance was characterized by poor to moderate reliability and markedly higher within‐subject variability. This clear task‐dependent contrast supports the notion that more challenging balance tasks provide a stronger and more stable signal for quantifying postural control, even when assessed using portable, non‐laboratory‐grade instrumentation. Importantly, these findings suggest that, in healthy young adults, the reliability of postural sway measures obtained with a portable low‐cost force plate is strongly influenced by task selection. Single‐leg stance may provide sufficient postural demand to improve the stability of displacement‐ and velocity‐based CoP outcomes in this population. However, these results should not be generalized directly to clinical or rehabilitation populations without further validation.

The consistently higher inter‐session reliability observed in single‐leg stance compared with bipedal stance, aligns with the premise that more challenging postural tasks provide more robust differentiation of inherent balance capability. This pattern is clearly demonstrated in recent research with identical settings, which reported good to excellent reliability for single‐leg stance, with velocity measures showing particularly high consistency (Kozinc et al. 2025); ICC > 0.85 for velocity‐based measures in healthy young adults using standardized eyes‐open conditions). The present results also highlight differences across CoP parameters. Global descriptors such as total path length and mean CoP velocity (ICCs > 0.85) emerged as the most stable indicators, with sway velocity showing particularly favorable absolute error (CV ≈ 8%), in line with previous reliability studies (Salavati et al. 2009; Meshkati et al. 2011) which reported ICC values of 0.80–0.90 for velocity‐based measures in young male athletes during bipedal stance with eyes‐open conditions, though without a standardized fixed visual target). In contrast, the CoP ellipse area and standard deviation of sway demonstrated considerably lower reliability, consistent with a previous study (ICC = 0.60–0.75 for ellipse area in healthy young adults using a fixed visual target at eye level; Santos et al. 2008). The high variability of these measures reflects their susceptibility to day‐to‐day variation, potentially due to sporadic corrective movements. This observation is supported by evidence showing that the sway area has the highest variability (CV ≈ 19.5%) among all measured parameters. Finally, the trend toward higher ICCs in the right leg across multiple outcomes points to the possible influence of limb dominance and task‐specific strategies on single‐leg performance (Kozinc and Šarabon 2021; Kozinc et al. 2025). Although our study did not directly assess this, it highlights the need for limb‐specific reporting in future research using single‐leg stance.

Bipedal stance demonstrated poorer reliability across all center‐of‐pressure parameters under the present conditions, suggesting limited usefulness for longitudinal assessment in healthy young adults when using this device–software setup. However, this finding should not be interpreted as evidence that bipedal stance is inherently unsuitable for postural assessment. In young healthy adults, the low task demands of bipedal quiet standing may restrict sway magnitude and reduce between‐subject variability, thereby lowering ICC values and increasing the relative influence of measurement noise. In populations with greater balance impairment, such as older adults or individuals with neurological or musculoskeletal disorders, bipedal stance may still provide clinically relevant information. Higher reliability during bipedal standing has been reported in mixed samples of athletes and non‐athletes, particularly under eyes‐closed conditions (Meshkati et al. 2011). Therefore, in the present sample of healthy young adults, bipedal stance appeared less suitable than single‐leg stance for repeated assessments aimed at detecting individual differences over time. Nevertheless, its usefulness should be evaluated separately in populations with greater postural instability, where larger sway amplitudes may improve discriminative ability and reliability.

No previous study has examined the same force plate model, but other evidence with different models further corroborates the task‐ and metric‐dependent nature of postural sway reliability. Schröder et al. (2024) evaluated a portable pressure plate against laboratory‐grade force plates during quiet standing and reported good‐to‐excellent test–retest reliability for velocity‐based CoP measures (ICC ≥ 0.80), whereas amplitude‐ and area‐based metrics as well as coordination measures such as BLS showed substantially lower reliability. Despite strong correlations between instruments for several outcomes, the authors identified a systematic proportional bias, with the pressure plate consistently underestimating CoP magnitude relative to force plates, particularly at higher sway levels. These findings closely parallel both the observations of Golriz et al. (2012), who reported poor concurrent validity for sway area and magnitude‐dependent disagreement between devices, and the results of Walsh et al. (2021), who demonstrated that reliability and agreement improve under more challenging sensory conditions. Importantly, Schröder et al. emphasized that, despite acceptable reliability, cross‐device interchangeability is limited and that longitudinal assessments should be performed using the same device—a recommendation strongly supported by our results. Extending this literature, the present study demonstrates that even within a single portable force plate system, insufficiently demanding tasks such as quiet bipedal stance yield poor inter‐session reliability with healthy adults, whereas increased task difficulty (single‐leg stance) substantially enhances the stability of velocity‐ and displacement‐based metrics. Collectively, these findings indicate that reliable longitudinal assessment of postural control using portable devices is less constrained by platform type than by appropriate task selection and outcome choice, with velocity‐related CoP measures under challenging stance conditions emerging as the most robust indicators.

### 4.1 Implications for Physiotherapy Practice

Portable low‐cost force plates may support physiotherapy practice by enabling objective quantification of postural control outside specialized laboratories. However, the present findings should be interpreted as preliminary reliability evidence for healthy young adults only. The sample was relatively small, consisted mainly of men, and did not include older adults, patients undergoing rehabilitation, or individuals with known balance impairments. Therefore, the present results should not be used to infer clinical utility across physiotherapy populations without further validation. In this specific population, single‐leg stance appears preferable to bipedal quiet stance for repeated assessments, particularly when using total CoP displacement and displacement velocity as outcomes.

### 4.2 Limitations

The present findings should be interpreted in light of several methodological limitations. First, in healthy young adults, quiet bipedal stance may exhibit a restricted range of postural sway due to low task demands, which can limit the ability to discriminate subtle inter‐individual differences and amplify the relative influence of measurement noise or day‐to‐day strategy fluctuations. While it is plausible that the increased neuromuscular demand of single‐leg stance enhances signal relative to noise (thereby contributing to the higher reliability observed), this mechanism was not directly tested in the present study. Second, both stance paradigms are static and may have limited ecological validity, as they do not fully capture the dynamic balance demands encountered in daily life or sport. Third, although familiarization trials were provided, subtle habituation or strategy changes between sessions cannot be fully excluded. Several additional limitations should be acknowledged, including the homogeneous sample of healthy young adults, sex imbalance, absence of an a priori power analysis, lack of dynamic balance tasks and device comparison, and the potential inflation of reliability estimates due to trial averaging. An additional limitation is that raw force/load‐cell data were not independently processed by the investigators; therefore, filtering, CoP reconstruction, and velocity estimation could not be fully verified outside the manufacturer's proprietary software environment. The study also did not include simultaneous recordings from a laboratory‐grade force plate. Consequently, the findings do not establish criterion validity, measurement agreement, or interchangeability with laboratory‐based systems. They only indicate the extent to which the same device–software system produces consistent results across repeated sessions under the present testing conditions. An additional limitation is that raw force/load‐cell data were not independently processed by the investigators; therefore, filtering, CoP reconstruction, and velocity estimation could not be verified outside the manufacturer's proprietary software environment. This limits computational reproducibility and means that the reported reliability estimates apply to the complete device–software pipeline rather than to independently derived CoP variables. Future studies should, where possible, export raw force or load‐cell signals and compare manufacturer‐derived outputs with transparent, independently implemented processing routines.

## 5 Conclusions

This study demonstrates clear task‐dependent differences in the test–retest reliability of postural sway measures obtained using a portable low‐cost force plate in healthy young adults. Unipedal stance consistently exhibited higher reliability than bipedal quiet standing across all center‐of‐pressure parameters. In single‐leg conditions, total CoP displacement and displacement velocity showed good to excellent relative reliability alongside acceptable absolute error, supporting their use in repeated‐measures assessments in healthy young adults. In contrast, bipedal stance was characterized by poor to moderate reliability and substantially higher within‐subject variability, particularly for the ellipse area, suggesting limited suitability for longitudinal evaluation in healthy young adults under the present testing conditions. This conclusion should not be generalized to populations with greater balance impairment in whom bipedal stance may remain an appropriate and clinically meaningful assessment condition. Collectively, the results of this study suggest that reliability using portable systems may depend strongly on appropriate task selection and outcome choice, with more demanding stance conditions and global displacement‐ and velocity‐based parameters providing the most stable and interpretable measures. The clinical utility of these outcomes for monitoring rehabilitation progress or detecting meaningful changes in patient populations requires further investigation. The reported MDC_95_ values may help contextualize measurement error, but they should not be interpreted as thresholds for meaningful clinical improvement.

## Author Contributions

M.V., N.N. and Ž.K. conceived and designed the study. M.V. conducted data collection. Ž.K. supervised the study and contributed to methodological decisions. M.V. and Ž.K. performed data analysis and interpretation. M.V. and N.N. wrote the main manuscript draft. Ž.K. contributed to interpretation of results and critically revised the manuscript. All authors reviewed, edited, and approved the final version of the manuscript.

## Funding

The authors acknowledge the support by the Slovenian Research Agency through the research program KINSPO—Kinesiology for the effectiveness and prevention of musculoskeletal injuries in sports (P5‐0443). The funder played no role in study conceptualization, data collection and preparation of the manuscript.

## Ethics Statement

The study procedures were approved by the University of Primorska's Commission for Ethics in Human Subjects Research (approval number: 4264‐19‐6/23).

## Conflicts of Interest

The authors declare no conflicts of interest.

## Data Availability

Raw data pertaining to this study are freely available in the Zenodo database (https://doi.org/10.5281/zenodo.18399791).
